# Prevalence of Anxiety and Depression Among Patients With Type 2 Diabetes Mellitus in Eastern Region, Saudi Arabia: A Cross-Sectional Study

**DOI:** 10.7759/cureus.79490

**Published:** 2025-02-23

**Authors:** Ahmad K Alkhayyal, Ahmed S Almalki, Majed S Alsaeed, Sulaiman A Almedaires, Abdullatif A AlMuhaish, Sattam M Almikhlal, Ibrahim S AlKhalifah, Abdulrahman S Albujays

**Affiliations:** 1 Family Medicine, Ministry of National Guard - Health Affairs, Al-Ahsa, SAU; 2 Medicine, King Faisal University, Hofuf, SAU

**Keywords:** anxiety, depression, metabolic disorder, quality of life, stress, type 2 diabetes mellitus

## Abstract

Background: Type 2 diabetes mellitus (T2DM) is a chronic, progressive metabolic disorder frequently associated with depression and anxiety. It is characterized by elevated levels of glucose in the bloodstream. Individuals diagnosed with T2DM encounter an increased risk of various health complications, experience a low quality of life, and contribute to a significant economic burden due to healthcare costs associated with their condition.

Objective: The objective of this study was to evaluate the prevalence of anxiety and depression in patients with T2DM in the eastern region of Saudi Arabia, as well as to identify the factors that predict this comorbidity, with a focus on glycemic control, treatment adherence, and overall healthcare outcomes.

Methodology: A cross-sectional study was conducted with a sample size of 391 patients, including Saudi and non-Saudi individuals living in the eastern region of Saudi Arabia aged 18 years and older, all diagnosed with T2DM. Participants completed a self-administered online questionnaire that collected data on the sociodemographic background of the patients and their diabetic status. Scores such as the Patient Health Questionnaire-9 (PHQ-9) for depression and the Generalized Anxiety Disorder-7 (GAD-7) for anxiety, as well as their medication adherence, were covered in the final section. All analyses were performed using IBM SPSS Statistics for Windows, Version 26.0 (2019; IBM Corp., Armonk, New York, United States), and descriptive statistics were calculated for the study variables. Chi-square and exact probability tests were used to examine the associations between categorical variables.

Results: Most participants were aged 26-35 years (n=138, 35.3%), and were male (n=262, 67%) and Saudi nationals (n=365, 93.4%). A majority had diabetes for three to five years (n=128, 32.7%), with 196 (50.1%) having glycated hemoglobin (HbA1C) levels of 7-8%. Regarding physical activity, 134 (34.3%) participants reported no exercise and only 21 (5.4%) exercised daily. Additionally, anxiety symptoms were common, with 176 (45%) participants reporting some impact on daily tasks and 185 (47.3%) experiencing depression-related difficulties. Factors significantly associated with anxiety and depression included HbA1C levels, duration of diabetes, and the number of medications prescribed. Medication adherence challenges were prevalent, with 221 (56.5%) patients sometimes forgetting and 134 (34.3%) discontinuing their medication without consulting their physician.

Conclusion: Crucial factors associated with anxiety in our setting included being non-Saudi, having lower education levels, poor glycemic control, and the number of medications used. Depression, on the other hand, was found to be higher among unemployed patients, those with longer diabetes duration, poor glycemic control, and multiple medications. These findings emphasize the importance of healthcare providers in addressing both the medical and psychological needs of diabetic patients through tailored interventions to enhance their overall well-being.

## Introduction

Type 2 diabetes mellitus (T2DM) is one of the most prevalent chronic metabolic disorders, characterized by elevated blood glucose levels. Individuals diagnosed with T2DM face an increased risk of various health complications, reduced quality of life, and a significant economic burden due to healthcare costs associated with their condition [1]. In Saudi Arabia, T2DM is a widespread illness, with recent research indicating that 46% of individuals with T2DM experience anxiety and/or depression. Contributing factors include inadequate glycemic control, the presence of complications, and insufficient health support systems [2]. In 2017, global healthcare expenditure for individuals with diabetes was estimated at $850 billion. By 2018, more than 500 million people worldwide had T2DM. Although the prevalence of T2DM is currently similar in both high-income and low-income nations, predictions suggest a more pronounced increase in low-income countries in the future [3].

The World Health Organization (WHO) recognizes depression and anxiety as leading causes of disability worldwide, affecting approximately 300 million individuals [4]. Epidemiological studies have demonstrated that the correlation between T2DM and depression is twice as common as the occurrence of either condition alone [5]. Research has identified T2DM-related risk factors, such as insulin dependency, as significant contributors to the development of depression and anxiety. Insulin-dependent patients exhibit a higher incidence of depression and anxiety than their noninsulin-dependent counterparts [6]. As diabetes progresses and complications arise, particularly painful peripheral neuropathy and sexual dysfunction, the prevalence of depression increases significantly. Several studies have examined the prevalence of depression, anxiety, and/or stress among patients with T2DM in different regions [7]. For example, a study conducted in Arar, the northern region of Saudi Arabia, investigated this problem in 278 patients with T2DM aged 12 years and older [8]. The findings revealed that 127 (45.6%) patients experienced anxiety, 104 (37.4%) experienced depression, and 52 (18.7%) reported stress. Female patients were significantly more prone to depressive symptoms (n=74, 71.2%) (p = 0.043), whereas depression and anxiety were associated with older age, lower education levels, and being unmarried, which accounted for 48 (46.2%) of those with depression. Lower education levels were also linked to stress and difficulties in managing diabetes, contributing to psychological distress [8].

Moreover, the simultaneous presence of depression and anxiety in patients with T2DM presents a significant challenge for healthcare providers, as these conditions exacerbate one another [9,10]. Patients with both depression and anxiety have been found to have elevated HbA1c levels and greater fluctuations in blood glucose levels [11]. Consequently, individuals with long-term T2DM are more likely to experience higher rates of depression and/or anxiety, leading to poor disease management and reduced overall quality of life [12]. These findings highlight the need for healthcare providers to incorporate psychological evaluations into the care of patients with diabetes [13]. Nevertheless, studies investigating the prevalence of anxiety and depression among individuals with T2DM in Saudi Arabia remain scarce. Thus, this study aimed to address this gap by assessing the prevalence of anxiety and depression among patients with T2DM in the eastern region of Saudi Arabia, with a focus on glycemic control, treatment adherence, and overall healthcare outcomes.

## Materials and methods

This was a cross-sectional study conducted using a simple random sampling method to evaluate the prevalence of anxiety and depression in patients with T2DM in the eastern region of Saudi Arabia. Data were collected from December 31, 2024, to January 9, 2025. The study was approved by the Research Ethics Committee, King Faisal University (approval number: KFU-REC-2024-DEC- ETHICS2941 dated December 31, 2024). The study was carried out upon the acquisition of participant permission. Participation was voluntary and participants could withdraw at any time. No attempt was made to identify the subjects.

Inclusion and exclusion criteria

Inclusion criteria were patients with T2DM aged 18 years and older in the eastern region of Saudi Arabia. Individuals under the age of 18, without T2DM, outside the Eastern region of Saudi Arabia, and who did not complete the questionnaires were all excluded.

Sample size calculation

Epi Info software version 2.1 (Centers for Disease Control and Prevention (CDC), Atlanta, Georgia, United States) was used to calculate the sample size, keeping a 95% confidence interval (CI) and a 5% margin of error. It was determined that 385 was the minimum sample size, with a final sample size of 391 participants.

Data collection and scoring

A self-administered online survey was randomly shared over Telegram (Telegram Messenger Inc., Dubai, United Arab Emirates) and WhatsApp (Meta Platforms, Inc., Menlo Park, California, United States). The Patient Health Questionnaire-9 (PHQ-9), a validated depression screening tool, was used to measure depression. Depression was defined as a score of 5 or higher [14]. The Generalized Anxiety Disorder 7-item (GAD-7) scale was used to assess anxiety levels, defined as follows: 0-4 (minimal anxiety), 5-9 (mild anxiety), 10-14 (moderate anxiety), and 15-21 (severe anxiety) [15]. The Morisky Medication Adherence Scale-8 (MMAS-8), a validated screening tool, was used to measure medication adherence on a scale of 8 defined as follows: low adherence (score of <=0), medium adherence (score of 1 to <=2), and high adherence (score of 2>) [16].

Questionnaire

The questionnaire (See Appendices) included the sociodemographic background of the patients (age, gender, nationality, education level, marital status, employment, monthly income, and sports) in the first section of the questionnaire. Additionally, diabetic status (disease duration, last HbA1c, and number of drugs taken) was included in the second section. The PHQ-9 for depression and the GAD-7 for anxiety were included in the third section [14,15]. Finally, treatment adherence to medication questions such as forgetting to take their medication, skipping doses in the past two weeks for reasons other than forgetting, reducing or stopping medication without consulting a doctor due to feeling worse, forgetting medication while traveling, taking medication the previous day, and quitting medication when their health appeared to be under control) which was covered in the final section [16].

Validity and reliability

Ensuring validity and reliability is essential for producing accurate and generalizable findings. Content validity was established through expert review, where specialists in psychology, endocrinology, and diabetes education evaluated the questionnaire’s completeness and relevance. Additionally, face validity was confirmed via a pilot study with 30 participants, leading to minor modifications to improve clarity. Construct validity was examined through factor analysis, ensuring that each scale accurately reflected its respective construct. Furthermore, criterion-related validity was supported by comparing PHQ-9 scores with Beck’s Depression Inventory (BDI-II) and GAD-7 scores with the Hamilton Anxiety Rating Scale (HAM-A), confirming their alignment with well-established psychological assessments [14-16].

Reliability testing indicated strong internal consistency, with Cronbach’s alpha (α) values of 0.89 for PHQ-9, 0.92 for GAD-7, and 0.83 for MMAS-8, all exceeding the recommended threshold of 0.70. Test-retest reliability, assessed with 50 participants over two weeks, showed intraclass correlation coefficients (ICC) of 0.84 for PHQ-9, 0.83 for GAD-7, and 0.79 for MMAS-8, ensuring stability over time. Moreover, inter-rater reliability, measured using Cohen’s kappa (κ), exceeded 0.80, indicating strong agreement among raters. These findings confirm that the questionnaire demonstrates high validity and reliability, making it a robust tool for assessing depression, anxiety, and medication adherence among patients with T2DM.

Statistical analysis

Before conducting the analysis, data cleaning was performed to ensure the accuracy and consistency of the dataset. Any missing data were examined, and appropriate methods were applied to handle missing values, such as imputation or exclusion, depending on the extent of missing information. All analyses were performed using IBM SPSS Statistics for Windows, Version 26.0 (2019; IBM Corp., Armonk, New York, United States). Initially, descriptive statistics were calculated for the study variables. This involved generating frequency distributions and calculating percentages for categorical variables such as age, gender, nationality, educational level, marital status, job status, monthly income, and clinical factors like the duration of diabetes, glycated hemoglobin (HbA1C) levels, and the number of medications. Chi-square and exact probability tests were used to examine the associations between categorical variables to determine whether any significant relationship existed between the independent variables (e.g., demographic factors) and the dependent variables (anxiety and depression). A significance level of 0.05 (p-value < 0.05) was used as the threshold for determining statistical significance.

## Results

Demographic and lifestyle characteristics

Table 1 shows the demographic and lifestyle characteristics of the study participants. Most participants were aged 26-35 years (n=138, 35.3%), followed by those aged over 55 years (n=122, 31.2%), 36-55 years (n=118, 30.2%), and 18-25 years (n=13, 3.3%). The majority were male (n=262, 67.0%), while female patients accounted for 129 (33.0%). Most participants were Saudi nationals (n=365, 93.4%), with a minority being non-Saudi (n=26, 6.6%).

**Table 1:** Demographic characteristics of study participants (n=391) SR: Saudi Riyal

Characteristics	Frequency	Percentage
Age in years		
18-25	13	3.3%
26-35	138	35.3%
36-55	118	30.2%
> 55	122	31.2%
Gender		
Male	262	67.0%
Female	129	33.0%
Nationality		
Saudi	365	93.4%
Non-Saudi	26	6.6%
Educational level		
Below secondary	25	6.4%
Secondary education	107	27.4%
Bachelor's degree and above	259	66.2%
Marital status		
Single	120	30.7%
Married	230	58.8%
Divorced/widowed	41	10.5%
Employment		
Unemployed	123	31.5%
Student	41	10.5%
Employed	227	58.0%
Monthly income		
< 5000 SR	93	23.8%
5000-10000 SR	145	37.1%
> 10000 SR	153	39.1%
How many times did you exercise during the past four weeks?		
Never	134	34.3%
1-2 times/week	143	36.6%
3-4 times/week	93	23.8%
Daily	21	5.4%

Educational levels varied, with 259 (66.2%) having a bachelor's degree or higher, 107 (27.4%) having completed secondary education, and 25 (6.4%) with education below the secondary level. Marital status showed that the majority were married (n=230, 58.8%), while 120 (30.7%) were single, and 41 (10.5%) were divorced or widowed. Regarding employment, 227 (58.1%) were employed, 123 (31.5%) were unemployed, and 41 (10.5%) were students. Monthly income distribution revealed that 153 (39.1%) reported more than 10,000 Saudi Riyal (SR), 145 (37.1%) reported 5,000-10,000 SR, and 93 (23.8%) reported less than 5,000 SR.

Regarding physical activity, 143 (36.6%) exercised one to two times per week in the past four weeks, 134 (34.3%) did not exercise at all, 93 (23.8%) exercised three to four times per week, and 21 (5.4%) reported exercising daily.

Diabetes-related data of the study participants 

Table 2 summarizes the diabetes-related data of the study patients, focusing on the duration of T2DM. The largest proportion of patients had diabetes for three to five years (128, 32.7%), followed by those with a duration of less than 2 years (117, 29.9%). Patients with diabetes for 6-10 years accounted for 81 (20.7%), while those with a duration of more than 10 years constituted 65 (16.6%). For HbA1C levels in the last three months, 196 (50.1%) had levels between 7-8%, indicating suboptimal glycemic control. Additionally, 110 (28.1%) had HbA1C levels below 6.5%, while 71 (18.2%) had levels between 9-10%. A smaller subset, 14 (3.6%), had levels greater than 10%, reflecting poor glycemic control.

**Table 2:** Distribution of duration of type 2 diabetes mellitus, HbA1C levels, and number of medications among participants (N=391) HbA1C: glycated hemoglobin

Diabetes data	Frequency	Percentage
Duration of type 2 DM in years		
< 2	117	29.9%
3-5	128	32.7%
6-10	81	20.7%
> 10	65	16.6%
Level of HbA1C in last 3 months		
< 6.5%	110	28.1%
7-8%	196	50.1%
9-10%	71	18.2%
> 10%	14	3.6%
Number of received drugs		
1 drug	120	30.7%
2 drugs	121	30.9%
3 drugs	82	21.0%
4 drugs	42	10.7%
> 4 drugs	26	6.6%

In terms of the number of drugs received, most patients were on monotherapy (n=120, 30.7%) or dual therapy (n=121, 30.9%). A smaller percentage were on triple therapy (n=82, 21.0%), four drugs (n=42, 10.7%), or more than four drugs (n=26, 6.6%).

Frequency and impact of anxiety symptoms

Table 3 illustrates the frequency and impact of anxiety symptoms experienced by the study participants. Feeling nervous, anxious, or on edge was notably common, with 157 (40.2%) patients experiencing this symptom "Several days," 48 (12.3%) "More than half the days," and 36 (9.2%) "Nearly every day." Similarly, a substantial proportion of patients 125 (32.0%) reported being unable to stop or control worrying "Several days," while 56 (14.3%) and 21 (5.4%) experienced this "More than half the days" and "Nearly every day," respectively.

**Table 3:** Frequency of anxiety symptoms among study participants (N=391)

Anxiety items	Not at all		Several days		More than half the days		Nearly every day	
	Frequency	Percentage	Frequency	Percentage	Frequency	Percentage	Frequency	Percentage
Feeling nervous, anxious, or on edge.	150	38.4%	157	40.2%	48	12.3%	36	9.2%
Not being able to stop or control worrying.	189	48.3%	125	32.0%	56	14.3%	21	5.4%
Worrying too much about different things.	126	32.2%	153	39.1%	78	19.9%	34	8.7%
Having trouble relaxing.	157	40.2%	118	30.2%	74	18.9%	42	10.7%
Being so restless that it's hard to sit still.	212	54.2%	96	24.6%	67	17.1%	16	4.1%
Becoming easily annoyed or irritable.	136	34.8%	158	40.4%	65	16.6%	32	8.2%
Feeling afraid as if something awful might happen.	232	59.3%	95	24.3%	40	10.2%	24	6.1%

Worrying too much about different things affected 153 (39.1%) participants "Several days," 78 (19.9%) "More than half the days," and 34 (8.7%) "Nearly every day." Additionally, 118 (30.2%) had trouble relaxing "Several days," with 74 (18.9%) and 42 (10.7%) experiencing this difficulty "More than half the days" and "Nearly every day." Being so restless that it's hard to sit still impacted 96 (24.6%) patients "Several days," 67 (17.1%) "More than half the days,” and 16 (4.1%) "Nearly every day." Becoming easily annoyed or irritable was also a frequent issue, with 158 (40.4%) experiencing it "Several days," 65 (16.6%) "More than half the days," and 32 (8.2%) "Nearly every day." Lastly, feeling afraid as if something awful might happen was reported by 95 (24.3%) patients "Several days," 40 (10.2%) "More than half the days," and 24 (6.1%) "Nearly every day."

The impact of anxiety on daily activities

Table 4 presents the impact of anxiety on daily activities with 176 (45%) participants reporting that it made their daily tasks somewhat difficult, while 52 (13.3%) felt it was very difficult, and 14 (3.6%) found it extremely difficult.

**Table 4:** The impact of anxiety symptoms among study participants (N=391)

How difficult have these problems made it for you to do your work, take care of things at home, or get along with other people?	Not difficult at all		Somewhat difficult		Very difficult		Extremely difficult	
	Frequency	Percentage	Frequency	Percentage	Frequency	Percentage	Frequency	Percentage
	149	38.1%	176	45%	52	13.3%	14	3.6%

Depression symptoms

As for depression symptoms experienced by participants, many reported little interest or pleasure in doing things, with 118 (30.2%) experiencing this for several days and 38 (9.7%) more than half the days (Table 5). A significant portion also reported feeling down, depressed, or hopeless, with 170 (43.5%) experiencing it for several days and 52 (13.3%) more than half the days. Trouble sleeping, whether falling asleep or sleeping too much, was common, with 117 (29.9%) reporting this symptom for several days and 85 (21.7%) experiencing it more than half the days. Fatigue or low energy was also reported by 172 (44%) for several days and 60 (15.3%) for more than half the days. Poor appetite or overeating affected 116 (29.7%) for several days and 66 (16.9%) for more than half the days. Feelings of inadequacy, such as thinking one is a failure or has let the family down, were reported by 104 (26.6%) several days and 45 (11.5%) more than half the days. Trouble concentrating on tasks like reading or watching TV was experienced by 110 (28.1%) during several days with 62 (15.9%) more than half the days. Slowness in movement or speech was reported by 103 (26.3%) for several days and 42 (10.7%) for more than half the days. Regarding thoughts of self-harm or being better off dead, 68 (17.4%) reported this symptom for several days and 44 (11.3%) experienced it more than half the days.

**Table 5:** Frequency of depression symptoms among study participants (N=391)

Depression items	Not at all		Several days		More than half the days		Nearly every day	
	Frequency	Percentage	Frequency	Percentage	Frequency	Percentage	Frequency	Percentage
Little interest or pleasure in doing things.	212	54.2%	118	30.2%	38	9.7%	23	5.9%
Feeling down, depressed, or hopeless.	153	39.1%	170	43.5%	52	13.3%	16	4.1%
Trouble falling or staying asleep, or sleeping too much.	143	36.6%	117	29.9%	85	21.7%	46	11.8%
Feeling tired or having little energy.	101	25.8%	172	44.0%	60	15.3%	58	14.8%
Poor appetite or overeating.	185	47.3%	116	29.7%	66	16.9%	24	6.1%
Feeling bad about yourself, thinking you are a failure or have let yourself or your family down.	223	57.0%	104	26.6%	45	11.5%	19	4.9%
Trouble concentrating on things, such as reading the newspaper or watching television.	192	49.1%	110	28.1%	62	15.9%	27	6.9%
Moving or speaking so slowly that others have noticed, or being fidgety and unable to sit still.	227	58.1%	103	26.3%	42	10.7%	19	4.9%
Thoughts that you would be better off dead, or of hurting yourself.	262	67.0%	68	17.4%	44	11.3%	17	4.3%

The impact of depression symptoms on daily life

Table 6 demonstrates the impact of depression symptoms on daily life with 185 (47.3%) participants reporting that it made their daily tasks somewhat difficult, while 57 (14.6%) found it very difficult, and 11 (2.8%) found it extremely difficult.

**Table 6:** Impact of depression symptoms among study participants (N=391)

How difficult have these problems made it for you to do your work, take care of things at home, or get along with other people?	Not difficult at all		Somewhat difficult		Very difficult		Extremely difficult	
	Frequency	Percentage	Frequency	Percentage	Frequency	Percentage	Frequency	Percentage
	138	35.3%	185	47.3%	57	14.6%	11	2.8%

Range of anxiety and depression severity

**Figure 1:**
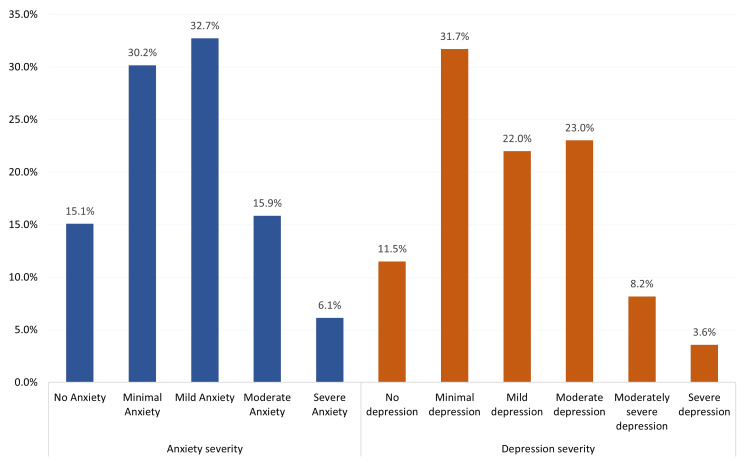
Prevalence of anxiety and depression among study participants (N=391)

Figure 1 shows a wide range of anxiety severity, with 59 (15.1%) of participants reporting no anxiety. A significant portion of the patients experienced minimal anxiety (n=118, 30.2%) and mild anxiety (n=128, 32.7%). Moderate anxiety was reported by 62 (15.9%), and 24 (6.1%) participants experienced severe anxiety. As for depression, 45 (11.5%) participants reported no depression, while 124 (31.7%) had minimal depression. Mild depression was experienced by 86 (22%), and moderate depression affected 90 (23%) participants. A smaller percentage had moderately severe depression (n=32, 8.2%) and severe depression (n=14, 3.6%).

Patient adherence to medication

Table 7 presents data on patient adherence to medication, highlighting various aspects of compliance and challenges faced. Regarding forgetting to take medication, 221 (56.5%) sometimes forgot, while 170 (43.3%) did not. In the past two weeks, 122 (31.2%) did not take their medication for reasons other than forgetting, whereas 269 (68.8%) consistently took their medication. When asked if they had ever reduced or stopped taking their medication without consulting their doctor because they felt worse when taking it, 134 (34.3%) responded affirmatively. Forgetting to bring medication when traveling or leaving home was reported by 166 (42.5%).

**Table 7:** Medication adherence and challenges among study participants (N=391)

Adherence Questions	Frequency	Percentage
Do you sometimes forget to take your medication?		
Yes	221	56.5%
No	170	43.5%
In the past 2 weeks, were there any days when you did not take your medication for reasons other than forgetting?		
Yes	122	31.2%
No	269	68.8%
Have you ever reduced or stopped taking your medication without consulting your doctor because you felt worse when taking it?		
Yes	134	34.3%
No	257	65.7%
When you travel or leave home, do you sometimes forget to bring your medication?		
Yes	166	42.5%
No	225	57.5%
Did you take your medication yesterday?		
Yes	317	81.1%
No	74	18.9%
When you feel that your condition is under control, do you sometimes stop taking your medication?		
Yes	117	29.9%
No	274	70.1%
Does taking medication daily ever bother you or create feelings of dissatisfaction?		
Yes	251	64.2%
No	140	35.8%
How often do you have difficulty remembering to take all your medications?		
Never/Rarely	118	30.2%
Occasionally	130	33.2%
Sometimes	106	27.1%
Usually	32	8.2%
Always	5	1.3%

Regarding whether patients took their medication the day before, 317 (81.1%) confirmed they did while 74 (18.9%) did not. About feeling that their condition was under control, 117 (29.9%) sometimes stopped taking their medication, whereas 274 (70.1%) continued their regimen. Feelings of dissatisfaction or bother due to daily medication intake were reported by 251 (64.2%), while 140 (35.8%) did not experience such feelings. As for remembering to take all medications, 118 (30.2%) rarely or never had difficulty, 130 (33.2%) occasionally had difficulty, 106 (27.1%) sometimes had difficulty, 32 (8.2%) usually had difficulty, and five (1.3%) always had difficulty.

Factors associated with anxiety and depression

Factors associated with anxiety and depression among the study participants are presented in Table 8. As for anxiety, there is a significant difference between Saudi and non-Saudi participants, with 26 (100%) non-Saudis reporting anxiety compared to 306 (83.8%) Saudis (p-value = 0.026). Anxiety prevalence also differs based on educational attainment. Among those with below-secondary education, 25 (100%) reported anxiety, compared to 94 (87.9%) of those with secondary education and 213 (82.2%) of those with a bachelor's degree or higher (p-value = 0.037). The level of HbA1C in the past three months significantly impacted anxiety. Among those with HbA1C levels below 6.5%, 85 (77.3%) reported anxiety, while 166 (84.7%) of those with levels of 7-8%, 68 (95.8%) with levels of 9-10%, and 13 (92.9%) with levels above 10% reported anxiety (p-value = 0.007). The number of drugs prescribed also appears to be a significant factor. Of patients taking one drug, 90 (75%) reported anxiety, while 107 (88.4%) of those taking two drugs, 69 (84.1%) of those taking three drugs, 40 (95.2%) of those taking four drugs, and 26 (100%) of those taking more than four drugs experienced anxiety (p-value = 0.001).

**Table 8:** Factors associated with anxiety and depression among study participants (N=391) P: Pearson X2 test; ^: Exact probability test; * P < 0.05 (significant)

Factors		Anxiety		Depression	
		Frequency	Percentage	Frequency	Percentage
Age in years	18-25	11	84.6%	10	76.9%
	26-35	119	86.2%	119	86.2%
	36-55	100	84.7%	107	90.7%
	> 55	102	83.6%	110	90.2%
X^2^; p-value		0.53; 0.950		1.25; 0.349	
Gender	Male	220	84.0%	231	88.2%
	Female	112	86.8%	115	89.1%
X^2^; p-value		1.36; 0.459		1.01; 0.772	
Nationality	Saudi	306	83.8%	320	87.7%
	Non-Saudi	26	100.0%	26	100.0%
X^2^; p-valu		4.25; 0.026*^		3.80; 0.057^	
Educational level	Below secondary	25	100.0%	25	100.0%
	Secondary education	94	87.9%	90	84.1%
	Bachelor's degree / above	213	82.2%	231	89.2%
X^2^; p-value		4.01; 0.037*		3.76; 0.068	
Marital status	Single	108	90.0%	103	85.8%
	Married	188	81.7%	204	88.7%
	Divorced / widow	36	87.8%	39	95.1%
X^2^; p-value		3.30; 0.105		3.15; 0.271	
Employment	Unemployed	106	86.2%	112	91.1%
	Student	37	90.2%	31	75.6%
	Employed	189	83.3%	203	89.4%
X^2^; p-value		3.01; 0.461		4.36; 0.022*	
Monthly income	< 5000 SR	82	88.2%	78	83.9%
	5000-10000 SR	121	83.4%	132	91.0%
	> 10000 SR	129	84.3%	136	88.9%
X^2^; p-value		2.98; 0.590		3.14; 0.235	
How many times did you exercise during the past 4 weeks?	Never	111	82.8%	117	87.3%
	1-2 times/week	123	86.0%	131	91.6%
	3-4 times/week	82	88.2%	82	88.2%
	Daily	16	76.2%	16	76.2%
X^2^; p-value		4.0; 0.457		4.77; 0.197	
Duration of type 2 diabetes mellitus in years	< 2	94	80.3%	94	80.3%
	3-5	110	85.9%	115	89.8%
	6-10	70	86.4%	78	96.3%
	> 10	58	89.2%	59	90.8%
X^2^; p-value		4.36; 0.367		6.35; 0.005*	
Level of HbA1C in last 3 months	< 6.5%	85	77.3%	83	75.5%
	7-8%	166	84.7%	183	93.4%
	9-10%	68	95.8%	66	93.0%
	> 10%	13	92.9%	14	100.0%
X^2^; p-value		9.01; 0.007*		12.60; 0.001*	
Number of received drugs	1 drug	90	75.0%	89	74.2%
	2 drugs	107	88.4%	115	95.0%
	3 drugs	69	84.1%	76	92.7%
	4 drugs	40	95.2%	41	97.6%
	> 4 drugs	26	100.0%	25	96.2%
X^2^; p-value		17.36; 0.001*		14.36; .001*^	

Considering depression, employment status played a significant role in depression. Among unemployed patients, 112 (91.1%) reported depression, while 31 (75.6%) students and 203 (89.4%) employed individuals experienced depression (p-value = 0.022). The duration of diabetes also significantly impacts depression levels. Among patients with diabetes for less than two years, 94 (80.3%) reported depression, compared to 115 (89.8%) of those with three to five years, 78 (96.3%) of those with 6-10 years, and 59 (90.8%) of those with diabetes for more than 10 years (p-value = 0.005).

Similarly, HbA1C levels influenced depression prevalence. Among patients with HbA1C levels below 6.5%, 83 (75.5%) reported depression, whereas 183 (93.4%) patients with levels of 7-8%, 66 (93.0%) with levels of 9-10%, and 14 (100%) with levels above 10% reported depression (p-value = 0.001). Additionally, the number of medications prescribed has a strong relationship with depression. Among patients taking one drug, 89 (74.2%) reported depression, while 115 (95.0%) of those taking two drugs, 76 (92.7%) taking three drugs, 41 (97.6%) taking four drugs, and 25 (96.2%) taking more than four drugs reported depression (p-value = 0.001).

## Discussion

Most study patients were young, with a large portion in middle age, and the majority were male and Saudi nationals. Educationally, many had at least a bachelor's degree, while exercise habits were poor, with over a third not engaging in any physical activity. Most patients had been living with T2DM for two to five years, and many showed suboptimal blood sugar control, as reflected by HbA1C levels of 7-8%. Most participants were on one to two medications, but a small percentage had poorly controlled diabetes requiring more intensive treatment. These findings highlight the need for improved lifestyle interventions and better clinical management strategies for T2DM in the region.

Concerning mental health (depression and anxiety), the current study showed that a substantial portion of the patients experienced varying degrees of anxiety, ranging from minimal to severe. The presence of anxiety in patients with diabetes is a well-documented phenomenon [17]. A study conducted by Ali et al. reported that anxiety levels in patients with diabetes are significantly higher compared to individuals without diabetes, attributing this to the stress of managing a chronic illness [18].

Depression was also prevalent among the study participants, with many experiencing mild to moderate levels of depression. This finding is consistent with international studies, which have shown that individuals with T2DM are at a higher risk of developing depression. For example, a study by Anderson et al. found that approximately twice as many individuals with diabetes suffer from depression as the general population [6].

In the Middle Eastern context, a study by Al-Amer et al. indicates that depressive symptoms are common among diabetes patients in Jordan, highlighting the need for integrated mental health services within diabetes care programs [19]. The comparison with studies from other regions underscores the global nature of the issue. For instance, the prevalence of anxiety and depression among diabetic patients in Dhahran, Saudi Arabia [20], aligns with our findings, emphasizing the widespread impact of these mental health conditions. Moreover, the comparative study between Saudi Arabia and Egypt by Mahmoud et al. illustrates similar trends, with higher depression rates observed in Saudi patients and higher anxiety rates in Egyptians (13 of 19 patients) [21]. This suggests that cultural, social, and healthcare system differences may influence the mental health outcomes of diabetes patients. The prevalence of depression among participants with T2DM in our study exceeded the rates reported in several local studies, which ranged from 36% to 40% [22-30]. However, one study conducted in secondary and tertiary hospitals found a significantly higher prevalence, with 172 (77.8%) diabetes patients experiencing depression [31], which closely matches the result of our study. Additionally, our study reports a higher prevalence of anxiety compared to the studies in the Eastern Region. This discrepancy may be attributed to differences in study populations, methodologies, and regional variations in healthcare access and support systems. 

This study identifies several factors contributing to anxiety and depression in T2DM patients, offering insights for targeted healthcare interventions. Non-Saudi patients reported higher anxiety levels, potentially due to additional factors such as cultural adjustment and socio-economic challenges. Lower education was associated with higher anxiety, suggesting that education may enhance coping mechanisms and health literacy. Poor glycemic control, indicated by elevated HbA1c levels, correlated with increased anxiety, consistent with prior research linking poor glycemic control to anxiety. Additionally, a higher number of prescribed medications was associated with greater anxiety, likely due to the complexity of managing multiple drugs and the severity of the disease. 

In terms of depression, unemployment was a significant predictor, with higher depression rates seen in unemployed individuals, possibly due to financial strain and social insulation. The duration of diabetes was also linked to higher depression rates, reinforcing the connection between chronic illness duration and psychological distress. Poor glycemic control was associated with higher depression, highlighting the bidirectional relationship between diabetes management and mental health. Furthermore, the number of medications prescribed was strongly associated with depression, underscoring the psychological burden of managing complex treatment regimens. Managing multiple medications often complicates a patient’s daily routine. The constant need to remember dosing schedules, deal with potential side effects, and worry about drug interactions can be overwhelming, which in turn may elevate stress levels and contribute to depressive symptoms.

Limitations of the study

This study has certain limitations. First, because certain participants may not have provided truthful answers to all questions, using a survey that they submitted via social media may have introduced inaccuracies in data. Second, while the sample size of our study is not small, a larger sample size provides a more accurate representation of the target population, which reduces the possibility of random fluctuations. The estimations obtained from the sample are therefore more likely to accurately represent the population's actual prevalence. Finally, the study measured drug usage by reporting only the number of drugs without detailing the types or dosages. This approach restricts the depth of the analysis, as it does not allow for an examination of how specific drug types or dosages may differentially impact the outcomes.

## Conclusions

The study highlights the significant burden of anxiety and depression among T2DM patients in the eastern region of Saudi Arabia. Healthcare providers should be aware of the psychological challenges faced by diabetes patients and offer appropriate support and interventions to improve their overall well-being. As for factors associated with mental health disorders, non-Saudis, individuals with lower education levels, and patients with poor glycemic control and multiple medications are at higher risk for anxiety. Similarly, depression is more prevalent among unemployed patients, those with longer diabetes duration, and those with poor glycemic control and multiple medications. These findings underscore the need for tailored interventions that address both the medical and psychological aspects of diabetes care to improve overall well-being. Further research is needed to assess the prevalence of depression and anxiety in different regions of Saudi Arabia.

## Appendices

Questionnaire

Do you agree to participate in the survey? *

Yes

Section 1: Personal Information

What is your gender? *

- Male

- Female

What is your age? *

- Under 18 years

- 18-25 years

- 26-35 years

- 36-55 years

- Over 55 years

What is your nationality? *

- Saudi

- Non-Saudi

What is your educational level? *

- No formal qualification

- Elementary

- Intermediate

- Secondary

- Bachelor's or higher

What is your employment status? *

- Student

- Employed

- Unemployed

What is your marital status? *

- Single

- Married

- Divorced

- Widowed

What is your monthly income? *

- Less than 5,000 SAR

- 5,000 to 10,000 SAR

- More than 10,000 SAR

How often have you exercised in the past 4 weeks? *

- Never

- Twice a week

- 3-4 times a week

- Daily

Have you been diagnosed with Type 2 diabetes? *

- Yes

How long ago were you diagnosed with Type 2 diabetes (in years)? *

- 1 year or less

- 3-5 years

- 6-10 years

- More than 10 years

What was your last HbA1c level (past 3 months)? *

- 6.5 or less

- 7 to 8

- 9 to 10

- Higher than 10

How many medications are you taking? *

- 1

- 2

- 3

- 4 or more 

Section 2: Generalized Anxiety Scale (Please choose the option that best describes your condition).

Over the past 4 weeks, how often have you been bothered by any of the following problems? *

Not at all.    Several days.     More than half the days.     Nearly every day 

- Feeling nervous, anxious, or on edge.

- Not being able to stop or control worrying.

- Worrying too much about different things.

- Having trouble relaxing.

- Being so restless that it's hard to sit still.

- Becoming easily annoyed or irritable.

- Feeling afraid as if something awful might happen.

How difficult have these problems made it for you to do your work, take care of things at home, or get along with other people? *

- Not difficult at all

- Somewhat difficult

- Very difficult

- Extremely difficult

Section 3: Depression Measurement Tool (Please choose the option that best describes your condition).

Over the last 2 weeks, how often have you been bothered by any of the following problems? *

Not at all.    Several days.    More than half the days.    Nearly every day

- Little interest or pleasure in doing things.

- Feeling down, depressed, or hopeless.

- Trouble falling or staying asleep, or sleeping too much.

- Feeling tired or having little energy.

- Poor appetite or overeating.

- Feeling bad about yourself, thinking you are a failure or have let yourself or your family down.

- Trouble concentrating on things, such as reading the newspaper or watching television.

- Moving or speaking so slowly that others have noticed, or being fidgety and unable to sit still.

How difficult have these problems made it for you to do your work, take care of things at home, or get along with other people? *

- Not difficult at all

- Somewhat difficult

- Very difficult

- Extremely difficult

Section 4: Adherence to Diabetes Medication Scale (Please choose the option that best describes your condition).

Do you sometimes forget to take your medication? *

- Yes

- no 

In the past 2 weeks, were there any days when you did not take your medication for reasons other than forgetting? *

- Yes

- no 

Have you ever reduced or stopped taking your medication without consulting your doctor because you felt worse when taking it? *

- Yes

- no 

When you travel or leave home, do you sometimes forget to bring your medication? *

- Yes 

- no 

Did you take your medication yesterday? *

- Yes 

- no

When you feel that your condition is under control, do you sometimes stop taking your medication? *

- Yes

- no

Does taking medication daily ever bother you or create feelings of dissatisfaction? *

- Yes. 

- no

How often do you have difficulty remembering to take all your medications? *

- Never/Rarely

- Occasionally

- Sometimes

- Usually

- Always
